# To Predict the Prognosis and Immunological Characteristics of Pancreatic Cancer Based on Disulfide-Death Gene Death-Related lncRNA

**DOI:** 10.3390/biomedicines13040924

**Published:** 2025-04-09

**Authors:** Zhihong Liao, Tianxing Dai, Feng Yuan, Kai Li, Guoying Wang

**Affiliations:** Department of Hepatobiliary Surgery, The First Affiliated Hospital of Guangzhou Medical University, Guangzhou 510000, China; lzh1322603419@163.com (Z.L.); daitx1991@126.com (T.D.); yuanfeng1110@126.com (F.Y.); likai37@mail3.sysu.edu.cn (K.L.)

**Keywords:** pancreatic cancer, LncRNA, disulfide-death, prognosis

## Abstract

**Background:** Disulfide-dependent cell death, known as disulfide death, plays a pivotal regulatory role in the onset and progression of various cancers including pancreatic cancer. Despite its significance, little attention has been given to the study of disulfide death-related long non-coding RNAs (lncRNAs) in pancreatic cancer development and progression. **Methods:** This study utilized data from the Cancer Genome Atlas Project (TCGA) to analyze the transcriptome of pancreatic cancer. Co-expression analysis of genes associated with disulfide death was performed and six lncRNAs closely linked to disulfide death were identified through univariate and multivariate analysis. These lncRNAs were used to develop clinical prognostic models. The prognostic value of this model was then analyzed and further investigations included pathway enrichment analysis, tumor mutation load analysis, immune cell infiltration analysis, analysis of the tumor microenvironment (TME), and drug sensitivity analysis. **Results:** The developed prognostic model based on disulfide-associated lncRNAs exhibited significant prognostic value, allowing for reliable predictions of patient outcomes in pancreatic adenocarcinoma (PAAD). The analysis revealed that the six identified lncRNAs serve as independent prognostic factors, significantly correlating with patient survival and recurrence rates. Additionally, findings indicated notable differences in immune cell infiltration and drug sensitivity between high-risk and low-risk patient groups, suggesting potential therapeutic targets for enhancing treatment efficacy. **Conclusions:** Our findings revealed six disulfide death-associated lncRNAs with independent prognostic value, offering a crucial indicator for predicting the prognosis of pancreatic adenocarcinoma (PAAD) patients. Additionally, the analysis of tumor immune invasion and drug sensitivity provides a novel avenue for controlling tumor invasion and metastasis as well as reducing drug tolerance.

## 1. Introduction

Pancreatic cancer is a highly malignant tumor with a poor prognosis [1]. Detecting pancreatic cancer in its early stages is challenging due to the absence of obvious symptoms, leading to early metastasis, and as a result, patients are frequently diagnosed at advanced stages, with only a small minority eligible for radical surgery [2]. Recent studies have suggested that adjuvant chemotherapy can enhance the prognosis of pancreatic cancer patients by improving disease-free survival and overall survival [3]. However, the lack of effective biomarkers for early tumor diagnosis and the uncertain prognosis analysis present significant challenges for pancreatic cancer [4]. Thus, finding an effective biomarker or tool to predict treatment response and prognosis has become crucial.

Recently, a study revealed that intracellular sulfide accumulation in solute carrier family 7 member 11 (*SLC7A11*)-overexpressing cells, under glucose starvation, triggers cytoskeletal protein disulfide stress. This stress leads to actin network collapse and cell death, which is a new form of cell death known as disulfide death [5]. Unlike other cell death methods, this process has unique attributes. By inhibiting glucose transporters, cancer cells with overexpressed *SLC7A11* can cause this disulfide reaction, which inhibits tumor cell growth. Disulfide death-related genes have been shown to predict immune response and prognoses in hepatocellular carcinoma [6]. *SLC7A11* is often aberrantly expressed in multiple cancer types, including pancreatic adenocarcinoma (PAAD) which implies that the regulation of tumor cell growth may be accomplished through the upregulation of *SLC7A11* to cause disulfide death [7]. Nevertheless, the role of disulfide death in the development and progression of pancreatic cancer has yet to be systematically studied.

Long non-coding RNAs (lncRNAs) are a type of non-coding RNA that are more than 200 nucleotides long [8,9]. They have been found to play important roles in various cellular processes depending on their cellular localization. LncRNAs can be found in both the nucleus and cytoplasm of cells. In the nucleus, lncRNAs regulate genes by influencing cellular epigenetics and transcription levels [10]. They can be involved in mechanisms such as gene imprinting, histone modification, chromatin remodeling, transcriptional activation, transcriptional interference, nuclear transport, and cell cycle regulation [11,12]. In the cytoplasm, lncRNAs primarily regulate gene expression at the transcriptional and translational levels. They can affect various cellular processes related to cell growth, apoptosis, ferroptosis, and programmed cell death through their interactions with other molecules [13,14]. Studies have shown that lncRNAs are associated with disulfide-related cell death in lung cancer, cervical cancer, and colon cancer [15,16,17]. Additionally, lncRNAs have been identified as potential predictors for the diagnosis, prognosis, and drug sensitivity of PAAD [18,19]. However, the specific mechanism by which disulfide-related lncRNAs contribute to pancreatic cancer is still unclear.

## 2. The Present Study

### Synopsis

In this study, we utilized TCGA (https://portal.gdc.cancer.gov/, accessed on 4 April 2025) to obtain transcriptome data of pancreatic cancer. We performed the co-expression analysis of disulfide-related genes and conducted univariate and multivariate analysis to identify six long non-coding RNAs (lncRNAs) that were closely associated with disulfide-related cell death. These six lncRNAs were then used to construct a clinical prognosis model. We analyzed the prognostic value of this model and further conducted pathway enrichment analysis, tumor mutation burden analysis, immune cell infiltration analysis, tumor microenvironment (TME) analysis, and drug sensitivity analysis to gain more insights into the potential mechanisms underlying pancreatic cancer. The ultimate objective of this study was to provide guidance for prognosis analysis and the identification of drug treatment targets in pancreatic cancer by utilizing the constructed disulfide-related lncRNA prognostic model and the findings from subsequent analyses.

## 3. Materials and Methods

### 3.1. TCGA Data Collection

In this study, the researchers utilized the TCGA database to obtain transcriptome and clinical data for a cohort of pancreatic cancer patients. They employed the Perl programming language (version 5.40.1.) to extract lncRNA expression profiles from the transcriptome data and preprocessed the clinical data to ensure completeness. The resulting dataset, which comprised transcriptome and clinical information from 178 pancreatic cancer patients, included details such as gender, age, tumor TNM stage, treatment information (including whether patients underwent resection or received Folfirinox therapy), overall survival (OS), recurrence-free survival (RFS), and survival status. With this comprehensive dataset, we proceeded to construct a prognostic model focusing on disulfide-related lncRNAs and conducted subsequent analyses to investigate their potential impact on patient outcomes.

### 3.2. Expression Extraction of Disulfide-Death Genes and Analysis of Their Co-Expressed lncRNA

In this study, the expression of disulfide-death genes was extracted using the “limma” package of the R (Version: 4.3.1) programming language, which is commonly used for differential expression analysis to identify significantly differentially expressed genes in pancreatic cancer patients compared with healthy controls. After identifying these genes, the researchers performed co-expression analysis between them and the lncRNAs, using the Pearson correlation to measure the strength of association and applying selection criteria of corFilter = 0.4 and pvalueFilter = 0.001 to identify significant correlations. Finally, a sankey diagram was drawn to visually represent these co-expression relationships, aiding in the identification of potentially important regulatory relationships between disulfide-death genes and lncRNAs in pancreatic cancer. Subsequently, we optimized the model by multiple regression analysis and identified six lncRNAs (AC025048.4, AC074099.1, AC092171.5, LINC00519, LINC02004, and AC053503.1) as independent prognostic factors. Based on each independent prognostic lncRNA, we calculated the risk score as follows:

Risk score = (AC025048.4 × (−2.11091886092321)) + (AC074099.1× 0.894289468992622) + (AC092171.5 × (−0.864410736119382)) + (LINC00519 × 0.824896350145744) + (LINC02004 × 1.24561874202034) + (AC053503.1 × (−1.07460871624716)).

### 3.3. Prognostic Model Construction and Validation

In this study, using the PAAD dataset, we divided the dataset into a training set and a test set at a 1:1 ratio using the “caret” package in R (Version: 4.3.1). This random division ensured that both sets were representative of the overall dataset and reduced the risk of bias. Next, we performed univariate analysis of lncRNAs in the dataset to identify those potentially related to prognosis, assessing the association between each lncRNA expression and survival outcomes such as overall survival (OS) and disease-free survival (DFS). To further refine the selection of lncRNAs related to prognosis, the researchers employed the LASSO regression algorithm analysis, a regularization method that aids in feature selection and reduces model complexity [20]. This analysis helped identify the most relevant lncRNAs for constructing a prognostic risk model. In multivariate analysis, the selected lncRNAs were combined to construct the final prognostic risk model, which assigns a risk score to each patient based on the expression levels of these lncRNAs. Patients were then divided into high-risk and low-risk groups based on the median risk score. The researchers assessed the clinical prognostic value of the model by performing OS and DFS analysis on these groups, comparing the survival outcomes to evaluate the association between the risk score and patient prognosis. To evaluate the prognostic accuracy of the model, receiver operating characteristic (ROC) curve analysis and C-index curve analysis were used; the ROC curve assesses the sensitivity and specificity in predicting the patient survival status, while the C-index measures concordance between the predicted and observed survival times. Overall, these analyses aimed to identify a prognostic risk model based on lncRNA expression, determine its clinical value, and assess its accuracy in predicting patient outcomes. This study ultimately aimed to explore the prognostic and predictive value of disulfide-related lncRNAs in pancreatic cancer, providing research support for the discovery of potential biomarkers and personalized therapeutic targets in this disease.

### 3.4. Functional Enrichment Analysis

We utilized the R (Version: 4.3.1) packages “clusterProfiler”, “org.Hs.eg.db”, “circlize”, and “dplyr” to conduct GO pathway enrichment analysis, KEGG pathway enrichment analysis, and GSEA pathway enrichment analysis of the differential genes present in the high- and low-risk groups. Additionally, we employed the “ggplot2”, “enrichplot”, and “RColorBrewer” packages for visualizing the pathway enrichment. For the screening criterion, we considered *p* < 0.05.

### 3.5. Immune Cell Infiltration Analysis and Mutation Load Analysis

We applied the “CIBERSORT” algorithm to examine the differentiation of the immune cell microenvironment (TME) and immune cell function [21]. To visualize the results, we utilized the “ggpubr” package. We further analyzed the differential immune mutation load of risk genes using “limma” and employed the R (Version: 4.3.1) packages “survival” and “survminer” to explore the survival differences between the high- and low-risk groups of gene mutations.

### 3.6. Immunotherapy Analysis and Drug Sensitivity Analysis

We obtained the PAAD tumor immune dysfunction and escape data (TIDE.CSV) from the http://tide.dfci.harvard.edu/ website (accessed on 4 April 2024). To differentiate between the high-risk and low-risk groups and TIDE score, we employed the “limma” package in R (Version: 4.3.1). Additionally, we utilized the “limma”, “oncoPredict”, and “parallel” packages to effectively screen and analyze the drug sensitivity of pancreatic cancer drugs.

## 4. Results

### 4.1. Extract Disulfide Death-Related LncRNAs with Prognostic Value for PAAD Patients

**Figure 1:**
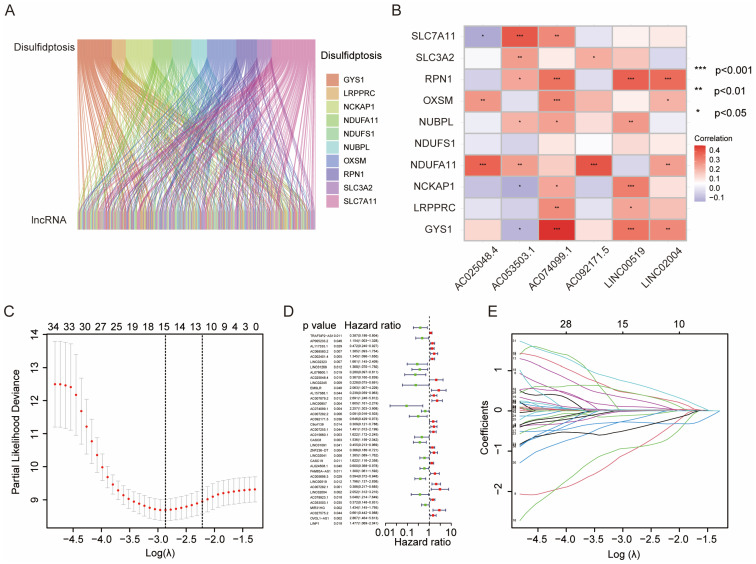
The comprehensive analysis of the disulfide-related genes and lncRNAs. (**A**) Visualization of the co-expression of disulfide-related genes and disulfide-related lncRNA in a Sankey diagram. (**B**) Correlation heatmap: This figure illustrates the correlation between the disulfide death-related genes and disulfide death-related lncRNAs. Red indicates a positive correlation and blue indicates a negative correlation, with darker colors indicating a stronger correlation. (**C**) LASSO regression analysis utilized the lowest point to determine the optimal number of lncRNAs. (**D**) Forest plot: Univariate COX analysis, based on the *p* < 0.05 significance level, identified disulfide death-related lncRNAs with prognostic value. The lncRNAs were categorized into high- and low-risk groups based on their analysis coefficients, denoted by green and red, respectively. (**E**) Partial likelihood deviation of different quantitative variables: The horizontal axis represents the logarithmic value of the independent variable lambda, while the vertical axis represents the coefficient of the independent variable.

According to the criteria of |R| > 0.4 and *p* < 0.05, we extracted 245 lncRNAs associated with double sulfur death from the TCGA PAAD transcriptomic database and downloaded the expression data of 10 genes related to double sulfur death for analysis. We then performed expression analysis of the double sulfur death-related genes and lncRNAs in the form of a volcano plot (Figure 1A). Afterward, we conducted univariate Cox regression analysis on the extracted lncRNAs associated with double sulfur death to screen out 37 significant lncRNAs. We further conducted LASSO regression analysis to confirm the number of disulfide death-related lncRNAs. Finally, we optimized the model by multiple regression analysis and identified six lncRNAs (AC025048.4, AC074099.1, AC092171.5, LINC00519, LINC02004, and AC053503.1) as independent prognostic factors. The risk score of each sample was then calculated based on the expression of six lncRNAs (Figure 1C–E). The correlation heatmap also showed the relationship between the cuproptosis-related genes and lncRNAs (Figure 1B).

### 4.2. Construction and Validation of Disulfide Death-Related lncRNA Prognostic Model

**Figure 2:**
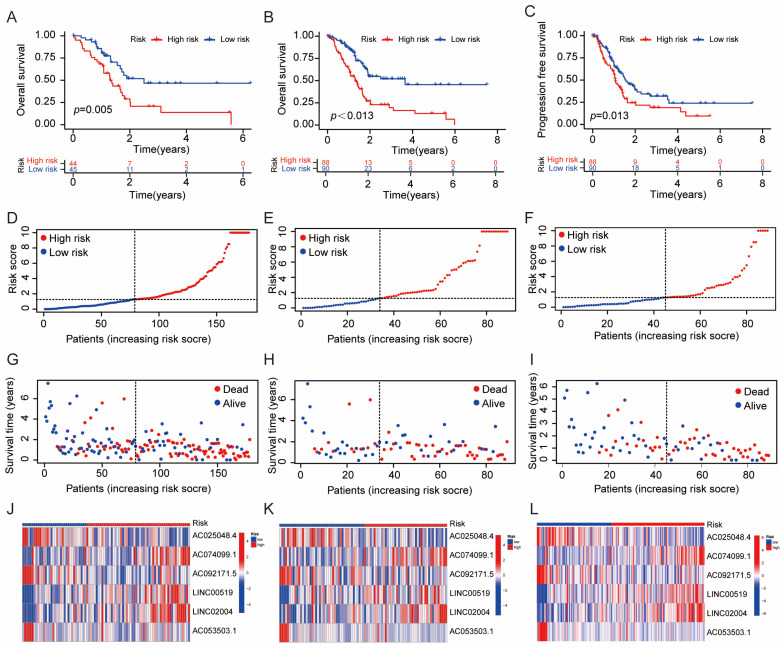
Survival and Risk Assessment of Disulfide lncRNAs in High- vs. Low-Risk Groups. (**A**,**B**) Survival analysis of the high-risk group and low-risk group of disulfide gene-related lncRNAs; (**C**) analysis of the disease-free recurrence period between high-risk group and low-risk group of disulfide gene-related lncRNAs; (**D**–**F**) risk curve of the training group and test group for the disulfide gene-related lncRNAs; (**G**–**I**) survival status distribution of disulfide gene-related lncRNAs in the training group and test group; (**J**–**L**) the expression levels of six characteristic lncRNAs in the high-risk group and low-risk group in the training group and test group.

To analyze the prognostic value of disulfide-related lncRNAs associated with disulfide death, we constructed a prognostic model using the screened lncRNA. These lncRNAs were significantly related to prognosis and used as risk features. The patients were then divided into a high-risk group and a low-risk group based on the median risk score. Survival analysis was performed on both groups in the training and test groups. Our findings showed that in both groups, the high-risk group had a significantly lower survival time compared with the low-risk group (Figure 2A,B). This indicates that patients in the high-risk group of disulfide-related lncRNA had a poorer prognosis. Furthermore, a further analysis of disease-free survival (DFS) in the high-risk and low-risk groups revealed that the recurrence time was significantly shorter in the high-risk group compared with the low-risk group (Figure 2C). Additionally, disulfide-related lncRNAs were found to be associated with tumor invasion and metastasis.

By analyzing the risk coefficients of the disulfide-related lncRNAs, we were able to generate specific risk curves for the high- and low-risk groups. These curves showed that as the risk score increased, the survival time of the patients decreased (Figure 2D–F).

### 4.3. Independent Prognostic Value of Disulfide Death-Related lncRNAs

**Figure 3:**
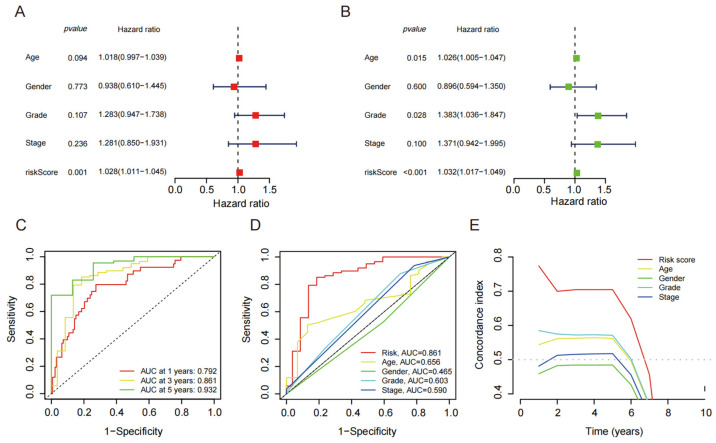
Analysis of Risk Score and Clinical Baseline Data through Univariate, Multivariate, ROC, and C-index Curves. (**A**) Univariate analysis of risk score and clinical baseline data; (**B**) multivariate analysis of risk score and clinical baseline data; (**C**) 1-, 3-, 5-year ROC curves of the risk score and clinical baseline data; (**D**) ROC curve of model risk score and clinical baseline data; (**E**) C-index curve of the risk score and clinical baseline data.

Based on the results of the univariate and multivariate Cox analyses, it was found that the risk score of the disulfide-related death lncRNA composition model had a significantly better prognostic value compared with other clinical characteristics (Figure 3A,B). These findings suggest that the disulfide-related lncRNAs have strong predictive power for patient prognosis. Furthermore, the ROC curve analysis demonstrated that the prognostic model based on the disulfide-related lncRNAs had excellent discriminative ability. The area under the ROC curve (AUC) at 1 year, 3 years, and 5 years was 0.792, 0.861, and 0.932, respectively (Figure 3C,D). These values indicate high accuracy of the model in predicting patient outcomes at different time points. Additionally, the C-index curve of the prognostic model also outperformed other clinical indicators, further highlighting its superior prognostic value. The C-index reflects the concordance between the predicted and observed survival probabilities, with a higher value indicating better predictive accuracy (Figure 3E). The fact that the prognostic model based on the disulfide-related lncRNAs had a significantly higher C-index compared with other clinical indicators suggests its robustness and reliability as a prognostic tool. Overall, these results demonstrate that the disulfide-related lncRNAs have strong prognostic value, and the prognostic model based on these lncRNAs exhibits excellent predictive accuracy for patient outcomes. It could serve as a valuable tool in assessing prognosis and making informed clinical decisions in patients with disulfide-related death.

### 4.4. Enrichment Analysis of lncRNA Related Pathways Related to Disulfide Mortality

**Figure 4:**
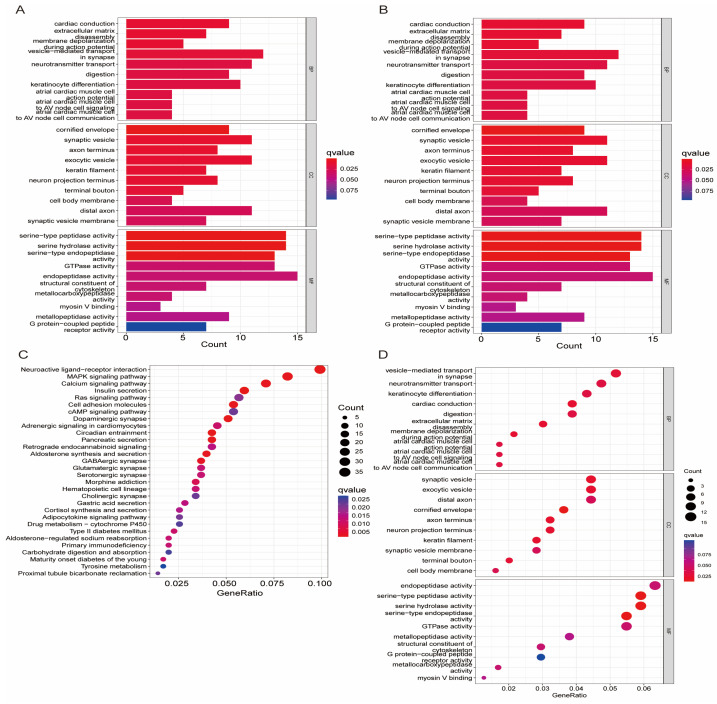
Enrichment Analysis of Disulfide Death-Related lncRNAs in GO and KEGG Pathways. (**A**,**B**) GO pathway enrichment analysis of disulfide death-related lncRNAs; (**C**,**D**) KEGG pathway enrichment analysis of disulfide death-related lncRNAs.

**Figure 5:**
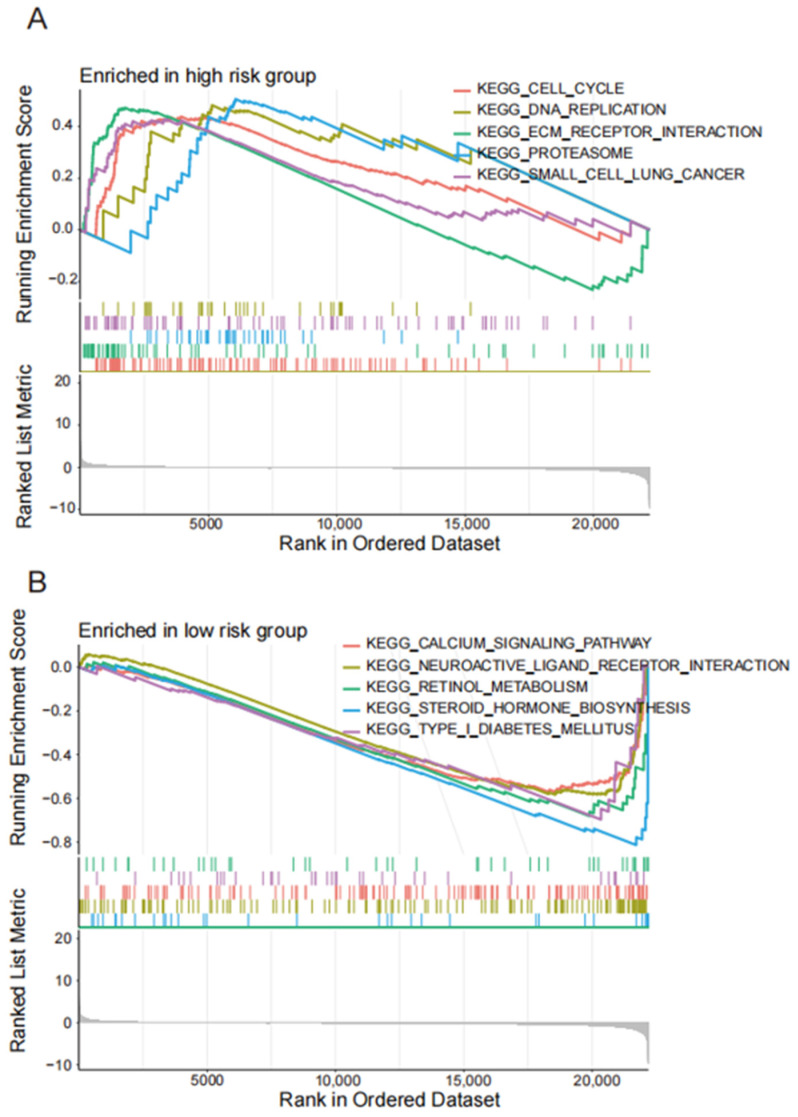
The disulfide death-related lncRNAs in GSEA pathway enrichment analysis. (**A**,**B**) GESA pathway enrichment analysis between the high-risk group and low-risk group.

The GO pathway enrichment analysis suggests that the disulfide-related lncRNAs were involved in serine-type peptidase activity, serine hydrolase activity, and serine-type endopeptidase activity (Figure 4A,B). These pathways are essential for the degradation and regulation of proteins in the cell. The KEGG pathway analysis revealed that the disulfide-related lncRNAs were mainly associated with neuroactive ligand–receptor interaction, MAPK signaling pathway, and the calcium signaling pathway (Figure 4C,D). These pathways play vital roles in cellular communication, signaling, and gene expression regulation. Moreover, the GSEA enrichment analysis further demonstrated that the high-risk group was enriched in cell cycle, DNA replication, ECM–receptor interaction, and proteasome pathways, which are closely related to cancer initiation, progression, and metastasis (Figure 5A,B). In contrast, the low-risk group was mainly associated with calcium signaling pathway, neuroactive ligand–receptor interaction, retinol metabolism, and steroid hormone biosynthesis pathways, which are essential for cellular homeostasis, metabolism, and signaling.

### 4.5. TME, Immune Cell Infiltration and Tumor Mutational Burden Analysis of Disulfide-Related lncRNA

**Figure 6:**
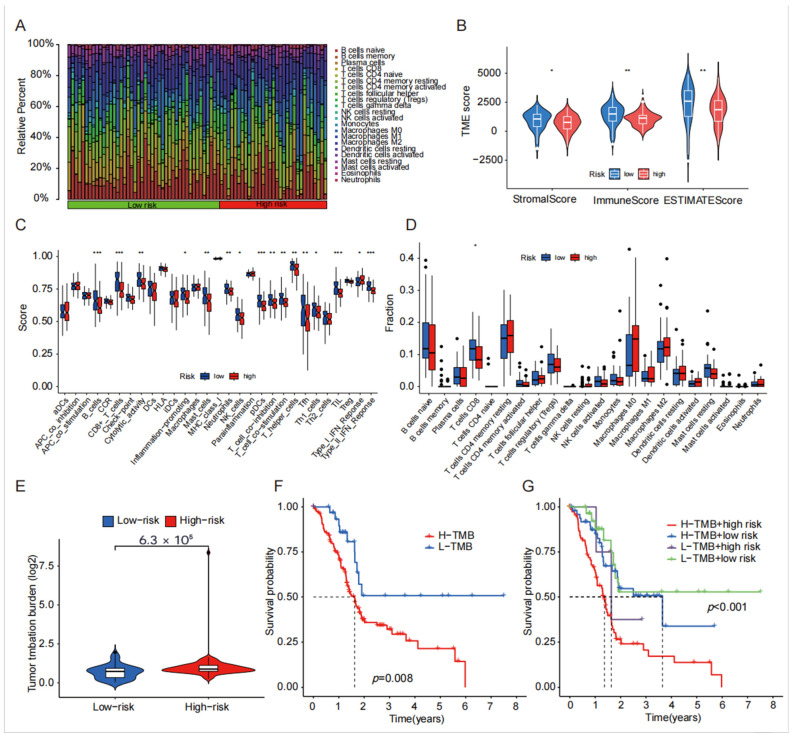
The differential analysis of cell scores, immune cell infiltration, and gene mutation burden with survival analysis in the high-risk vs. low-risk groups. (**A**) Differential analysis of the stromal cell, immune cell, and total cell line scores in the high-risk group and low-risk group; (**B**) differential analysis of immune cell infiltration between the high-risk group and low-risk group; (**C**) visualization of the distribution of immune cell infiltration in the high-risk group and low-risk group; (**D**) boxplot of functional differentiation analysis of immune cell infiltration in the high- and low-risk groups; (**E**) analysis of gene mutation burden in the high-risk group and low-risk group; (**F**,**G**) survival analysis between the high-risk group and low-risk group of gene mutation burden. (* indicates *p* < 0.05, ** indicates *p* < 0.01, *** indicates *p* < 0.001).

In the analysis of the immune microenvironment, we observed that the StromalScore, ImmuneScore, and ESTIMATEScore were higher in the low-risk group when compared with the high-risk group (Figure 6B). Notably, there were significant differences in all three immune scores between the high-risk and low-risk groups (*p* < 0.05). Moving on to the boxplot analysis of immune cell infiltration, we found a significant distinction between the high-risk and low-risk groups, primarily in terms of CD8 infiltration distribution. The analysis of immune cell function differentiation revealed significant differences between the high- and low-risk groups, specifically in the expression of B_cells, CD8+_T_cells, Type_II_IFN_Reponse, TIL, and pDCs (Figure 6A,C,D). Furthermore, the mutation burden analysis demonstrated a significantly higher mutation burden in the high-risk group compared with the low-risk group, indicating a greater predisposition to gene mutations in the high-risk group (Figure 6E). Subsequently, survival analysis was conducted, revealing a significantly shorter survival time in the high mutation burden group compared with the mutation burden group. When the survival analysis was combined with the high- and low-risk groups, it became apparent that both the high mutation burden and high-risk groups were crucial prognostic indicators of a poor prognosis (Figure 6F,G).

### 4.6. Drug Sensitivity Analysis of Disulfide Death-Related lncRNA in High- and Low-Risk Groups

**Figure 7:**
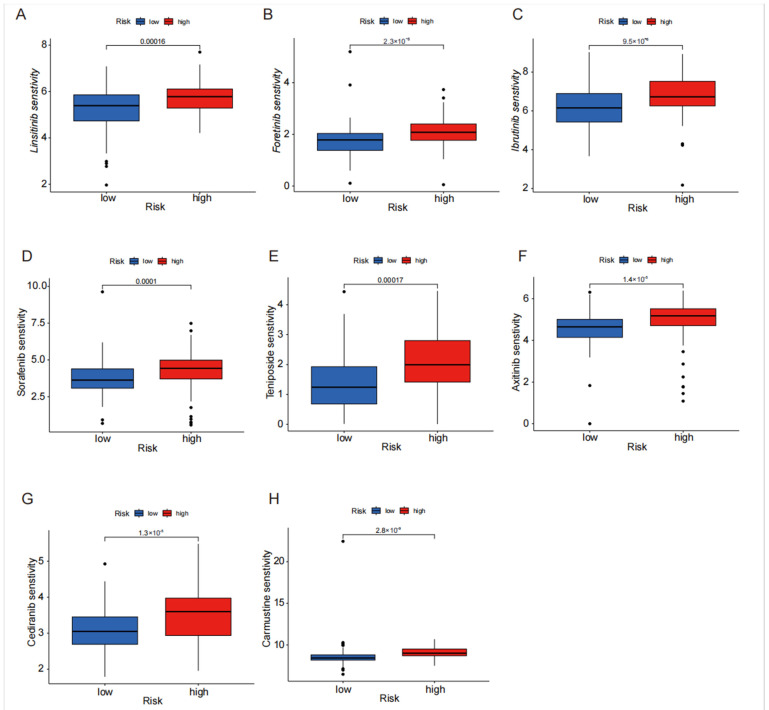
The differential sensitivity analysis of multiple drugs in high- vs. low-risk groups. (**A**–**H**) Differential sensitivity analysis of linitinib, foritinib, ibrutinib, sorafenib, tinipogan, axitinib, and carmustine in the high- and low-risk groups.

The disparity in sensitivity to the drug treatment between patients in the high-risk and low-risk groups was extensively examined utilizing the TIDE algorithm. The newly devised TIDE algorithm serves as a tool to assess the effectiveness of tumor immune checkpoint therapy. By analyzing drug sensitivity differences between the high-risk and low-risk groups, we can identify drugs that may have a greater potential for treating pancreatic cancer in some patients. Drugs such as sorafenib, ibrutinib, fruquintinib, and linifanib exhibit significantly higher sensitivity in the high-risk group compared with the low-risk group (Figure 7A–H), providing a predictive role for personalized medication in the treatment of pancreatic cancer. This personalized approach to medication can help improve the treatment outcomes and reduce the potential side effects by tailoring drug selection to an individual’s specific disease characteristics.

## 5. Discussion

Pancreatic cancer indeed poses significant challenges as it is often diagnosed at advanced stages, leading to limited treatment options and poor prognosis [22]. Although resection can significantly enhance the long-term survival in carefully selected patients, fewer than 20% of diagnosed cases are deemed suitable for this procedure, and individuals with resectable pancreatic cancer (PaC) who undergo resection exhibit considerably higher survival rates compared with those who are not candidates for surgery [23,24]. To address these limitations, identifying early diagnostic and prognostic indicators for pancreatic cancer is crucial. In this study, a model comprising six disulfide death-related lncRNAs was developed to potentially serve as such indicators. The model’s specificity was also validated, indicating its potential usefulness in diagnosing and predicting outcomes for pancreatic cancer patients. Early detection and accurate prognostication can enable timely intervention, personalized treatment strategies, and improved patient outcomes. Therefore, the development and validation of diagnostic and prognostic models are valuable contributions to pancreatic cancer research.

Indeed, long non-coding RNAs (lncRNAs) have emerged as key players in tumor development and progression [25,26,27]. Abnormal expression and mutations of lncRNAs can have significant effects on various aspects of tumor biology including growth, metastasis, and staging [28,29,30,31]. For example, the overexpression of lncRNA PSMB8-AS1 has been found to enhance the growth, invasion, and metastasis of pancreatic cancer cells by modulating the miR-382-3p/STAT1/PD-L1 pathway [32]. This suggests that targeting this lncRNA or its associated pathway may have therapeutic implications for pancreatic cancer. Similarly, the increased expression of lncRNA UCA1 has been shown to promote the invasion and metastasis of gastric cancer cells by acting as a sponge for miR-145, thereby influencing the expression of MYO6 [33]. These findings highlight the growing evidence supporting the potential of lncRNAs as valuable biological predictors and therapeutic targets in various cancers. Further research in this area may uncover additional lncRNAs with diagnostic, prognostic, and therapeutic significance, contributing to improved management strategies for cancer patients.

In this study, we identified 245 lncRNAs that were associated with disulfide death. Through univariate and multivariate analysis, we established and validated a signature lncRNA model that consisted of six lncRNAs closely associated with the prognosis of pancreatic cancer: AC025048.4, AC074099.1, AC092171.5, LINC00519, LINC02004, AC053503.1. The higher levels of LINC00519 in lung squamous cell carcinoma are associated with a worse outlook for patients, and research has also shown that LINC00519 may speed up the progression of the disease by interacting with miR-450b-5p and miR-515-5p, and by regulating a protein called YAP1 [34]. Previous research has reported that AC092171.5 is an m6A-related lncRNA, which holds potential as a biomarker for independently predicting the prognosis of pancreatic cancer [35]. Additionally, AC025048.4 is a CAF-related lncRNA and a prognostic marker associated with poor prognosis and immune infiltration in pancreatic cancer [36]. Kalantari et al. (2023) investigated the transcriptional profile of whole blood in early and metastatic stages of pancreatic cancer patients to identify potential diagnostic factors for early diagnosis [37]. Their findings provide further insights into the molecular mechanisms underlying pancreatic cancer progression and highlight the importance of blood-based biomarkers in early detection. The remaining lncRNAs have not been previously reported on, thus necessitating further studies to explore their roles in PAAD.

Our analysis categorized patients into high-risk and low-risk groups using the model and risk score calculations. The high-risk group demonstrated significantly reduced survival and recurrence times, with AUC values reaching 0.792, 0.861, and 0.932 at 1, 3, and 5 years, respectively, highlighting the prognostic significance of our risk score in pancreatic cancer. Furthermore, the results from the ROC, survival, nomogram, and heatmap analyses indicated that the prognostic features of the six disulfide-death lncRNAs were precisely distinguished between the high-risk and low-risk groups as well as between early-stage and late-stage patients. These lncRNAs served as reliable predictors of outcomes in patients with PAAD and were identified as prognostic factors independent of other common clinical characteristics. Moreover, through the gene set enrichment analysis (GSEA) of the lncRNAs, we made an important discovery: lncRNAs in the high-risk groups are significantly enriched in the proteasome pathway, strongly suggesting their specific involvement in the regulation of cell apoptosis and tumor progression in pancreatic cancer via this pathway. It has previously been reported that transcriptional lncRNAs can modulate gene expression through the proteasome pathway, which plays a crucial role in controlling various cellular processes, such as the cell cycle, transcription, and cell signaling, by degrading a wide range of proteins, thereby contributing to tumorigenesis and development [38,39]. For instance, the tumor suppressor RUNX3 inhibits tumor growth by promoting the degradation of oncogenic proteins through the ubiquitin-proteasome system [40].

The tumor microenvironment refers to the cellular environment surrounding tumor cells including immune cells, fibroblasts, endothelial cells, and mesenchymal cells [41]. This microenvironment plays a critical role in tumor development, growth, invasion, metastasis, and response to therapy [42,43,44]. In this model, the analysis of immune cell infiltration and differential expression revealed that the high-risk group had a lower number of CD8 cells, which may impair immune surveillance and promote tumor invasion and metastasis. CD8 T cells are important immune cells within the tumor microenvironment [45]. Various studies have demonstrated that the tumor microenvironment can suppress CD8 T cell activity, allowing tumor growth to proceed unchecked [46]. The decrease in CD8 T cell density within the tumor microenvironment has been identified as a negative prognostic marker for pancreatic cancer. Meanwhile, another critical player in the immunotherapy arena is the tumor mutational burden (TMB). The tumor mutational burden (TMB) represents the number of mutations per megabase of DNA sequenced in a specific cancer [47]. It was first identified as a potential biomarker for immune checkpoint inhibitor (ICI) therapy in melanoma [48]. TMB can also influence the efficacy of PD-L1 immunotherapy in non-small cell lung cancer [49]. lncRNAs are known to be abnormally expressed or mutated in various cancers [50,51,52]. According to the immune mutation burden analysis in this model, there was no statistically significant difference in immune mutation burden between the high-risk and low-risk groups. However, the high mutation burden group had a significantly lower survival time compared with the low mutation burden risk group. These findings suggest that the composition of the tumor microenvironment, specifically the presence of CD8 T cells, and the tumor mutational burden can impact the prognosis and response to therapy in pancreatic cancer. It highlights the importance of understanding the tumor microenvironment and genomic characteristics for developing effective treatment strategies.

In this paper, we subsequently utilized the pRRophetic algorithm to identify potential effective drugs for tumor immunotherapy and assessed the sensitivity of these agents, which are currently used in the treatment of various cancers including lung, lymphoma, pancreatic (PAAD), breast, kidney, and bile duct cancers. The analysis revealed significant differences in the sensitivity of tumor drugs between the high-risk and low-risk groups, and our findings indicated that high-risk patients demonstrate increased sensitivity to anticancer drugs. However, further investigation is necessary to elucidate the underlying drug mechanisms and their impact on the progression of PAAD. The mechanisms underlying lncRNA-mediated drug resistance involve the regulation of drug efflux, DNA damage repair, cell cycle, apoptosis, epithelial–mesenchymal transition (EMT), signal pathway induction, and angiogenesis [53]. The drug resistance role of lncRNAs has been validated in various tumor types. For example, HOTAIR has been shown to inhibit the expression of the P21 protein, thereby increasing the resistance to cisplatin in lung adenocarcinoma cells [54]. The overexpression of lncRNAs in drug-resistant gastric cancer cells has been found to reduce the degradation of ATG14 mRNA by sequestering MIR188-3p, leading to the activation of autophagy and chemotherapy immunity [55].

Despite the meaningful findings obtained in this study on predicting the prognosis and immune characteristics of pancreatic cancer based on disulfide bond-related long non-coding RNAs associated with cell death, several limitations remained. Due to the limited sample size, our results may lack generality, and the statistical significance may be affected. To address this issue, future studies could employ a larger sample size, encompassing a broader population of pancreatic cancer patients to enhance the representativeness and reliability of the results. Additionally, more advanced statistical methods, such as machine learning and deep learning algorithms, could be considered for processing and analyzing large datasets, thereby improving the efficiency and accuracy of statistical analyses. this study only focused on the prognostic value of disulfide bond-related lncRNAs in pancreatic cancer, without delving into their specific mechanisms of action during the onset and progression of the disease. To gain a more comprehensive understanding of the roles of these lncRNAs in pancreatic cancer, future research should further explore their interactions with pancreatic cancer-related signaling pathways, gene expression regulatory networks, and immune escape mechanisms. Through in-depth functional experiments and mechanistic studies, the specific roles of these lncRNAs in the onset and progression of pancreatic cancer can be revealed, providing a theoretical basis for the development of new therapeutic strategies. Our experimental design may have been inadequate in some aspects such as the lack of in vitro and in vivo experimental validation. To improve upon this, future studies could adopt more advanced experimental techniques, such as cell culture models, and animal models, to more accurately measure and assess the functions of these lncRNAs in pancreatic cancer. By comprehensively applying multiple experimental techniques and methods, the roles and potential applications of these lncRNAs in pancreatic cancer can be fully evaluated.

## 6. Conclusions

The identification of six disulfide-death lncRNAs with independent prognostic value and the construction of a prognostic model is indeed significant in providing indicators for predicting the prognosis of pancreatic adenocarcinoma (PAAD) patients. This model can serve as a valuable tool for assessing patient outcomes and tailoring treatment strategies. Additionally, the analysis of tumor immune infiltration and drug sensitivity can offer insights into the mechanisms underlying tumor invasion, metastasis, and drug resistance. This understanding can help in the development of novel approaches to control these processes. By identifying new targets, such as the screened lncRNAs, the study opens up possibilities for the development of targeted therapies and interventions to improve patient outcomes in PAAD.

## Data Availability

Data supporting the findings of this study are available from the respective authors upon reasonable request.
